# Elucidation of the genetic determination of body weight and size in Chinese local chicken breeds by large-scale genomic analyses

**DOI:** 10.1186/s12864-024-10185-6

**Published:** 2024-03-20

**Authors:** Jie Wang, Jie Liu, Qiuxia Lei, Zhihe Liu, Haixia Han, Shuer Zhang, Chao Qi, Wei Liu, Dapeng Li, Fuwei Li, Dingguo Cao, Yan Zhou

**Affiliations:** 1 Poultry Breeding Engineering Technology Center of Shandong Province, Poultry Institute, Shandong Academy of Agricultural Sciences, Jinan, Shandong 250023 China; 2 Jinan Key Laboratory of Poultry Germplasm Resources Innovation and Healthy Breeding, Jinan, Shandong 250023 China; 3 Shandong Animal Husbandry General Station, Jinan, 250023 China; 4 Sichuan agricultural university college of animal science and technology, Chengdu, 611130 China

**Keywords:** Chicken, GWAS, Body weight, Body size

## Abstract

**Background:**

Body weight and size are important economic traits in chickens. While many growth-related quantitative trait loci (QTLs) and candidate genes have been identified, further research is needed to confirm and characterize these findings. In this study, we investigate genetic and genomic markers associated with chicken body weight and size. This study provides new insights into potential markers for genomic selection and breeding strategies to improve meat production in chickens.

**Methods:**

We performed whole-genome resequencing of and Wenshang Barred (WB) chickens (*n* = 596) and three additional breeds with varying body sizes (Recessive White (RW), WB, and Luxi Mini (LM) chickens; (*n* = 50)). We then used selective sweeps of mutations coupled with genome-wide association study (GWAS) to identify genomic markers associated with body weight and size.

**Results:**

We identified over 9.4 million high-quality single nucleotide polymorphisms (SNPs) among three chicken breeds/lines. Among these breeds, 287 protein-coding genes exhibited positive selection in the RW and WB populations, while 241 protein-coding genes showed positive selection in the LM and WB populations. Genomic heritability estimates were calculated for 26 body weight and size traits, including body weight, chest breadth, chest depth, thoracic horn, body oblique length, keel length, pelvic width, shank length, and shank circumference in the WB breed. The estimates ranged from 0.04 to 0.67. Our analysis also identified a total of 2,522 genome-wide significant SNPs, with 2,474 SNPs clustered around two genomic regions. The first region, located on chromosome 4 (7.41-7.64 Mb), was linked to body weight after ten weeks and body size traits. *LCORL*, *LDB2*, and *PPARGC1A* were identified as candidate genes in this region. The other region, located on chromosome 1 (170.46-171.53 Mb), was associated with body weight from four to eighteen weeks and body size traits. This region contained *CAB39L* and *WDFY2* as candidate genes. Notably, *LCORL*, *LDB2*, and *PPARGC1A* showed highly selective signatures among the three breeds of chicken with varying body sizes.

**Conclusion:**

Overall this study provides a comprehensive map of genomic variants associated with body weight and size in chickens. We propose two genomic regions, one on chromosome 1 and the other on chromosome 4, that could helpful for developing genome selection breeding strategies to enhance meat yield in chickens.

**Supplementary Information:**

The online version contains supplementary material available at 10.1186/s12864-024-10185-6.

## Background

Over thousands of years, hundreds of chicken breeds have evolved through natural and artificial selection across different environments [1], leading to significant phenotypic variations in body size, plumage, egg color, and flying ability [2]. Chicken meat has already established itself as one of the most efficient protein sources, accounting for over 30% of global meat products and playing a critical role in food security worldwide [3]. Body weight (BW) is an important economic trait primarily determined by minorgenes that interact with functional genes and serve as molecular markers, which are extensively studied for their association with weight gain.

Genetic analysis and pattern recognition have proven to be useful in identifying the origin of specific breeds or revealing their characteristic traits [4]. A study of commercial broiler populations for selective sweeps revealed numerous loci involved in the selection for muscle mass [5]. Genetic variations present among various chicken breeds have been leveraged to portray specific traits in these breeds [6–10]. As a result, there have been extensive genomic studies on the genetic conditioning of domestic animals such as chickens. Notably, several genes associated with growth and carcass traits in chickens have been identified, including insulin-like growth factors (*IGFs*) [5, 11] and growth hormone secretagogue receptor (*GHSR*) [12], lysozyme (*LYZ*), melanocortin 4 receptor (*MC4R*), adhesion G protein-coupled receptor G6 (*ADGRG6*) [13], etc. Furthermore, insulin like growth factor 2 mRNA binding protein 1 (*IGF2BP1*) has been shown to correlate positively with breast muscle weight and body size in various animals [14–16]. A genome-wide association study (GWAS) in F2 progenies of star and silky black-bone chickens also revealed that the LIM domain binding 2 (*LDB2*) gene was responsible for BW at seven to twelve weeks and weight gain at six to twelve weeks [17]. However, expanding these studies to include more breeds and larger populations is necessary for deeper insights into this research field with current focus on BW and size traits.

The chicken quantitative trait locus (QTL) database (release 45) includes 4,776 QTLs related to growth traits in chicken, such as BW at different ages and average daily gain [18]. However, many of the QTLs, particularly those identified in previous studies, lack precise mapping, leading to broad confidence intervals that encompass several genes. Despite intensive research into the genetics of meat production traits, the knowledge of key genes causing significant phenotypic variations remains limited. This study used a large population of Wenshang Barred (WB) chickens (*n* = 596) and three other breeds (*n* = 50) to minimize the risk of biases. It also performed a systematic comparison of the whole genome and a GWAS to identify the genes and genomic regions responsible for BW and size. This study provides new insights into the genetics of chicken selection and promises to facilitate the development of techniques for breeding native chickens.

## Materials and methods

### Ethics statement

All handling and experimental procedures concerning the chickens used in this study were conducted following ARRIVE guidelines. Ethical approval was granted by Science Research Department of the Shandong Academy of Agricultural Sciences (SAAS) (Jinan, China), with the reference number 2,021,001.

### Birds and sample collection

A total of 596 WB chickens obtained from Jinqiu Agriculture and Animal Husbandry Co., Ltd. (Wenshang, Shandong, China) were used in this study. The chickens were raised in accordance with the breeding and management protocols for WB chickens. The experimental chickens remained in cages throughout the entire process, including the brooding stage from 0 to 7 weeks of age, the growing stage from 8 to 16 weeks of age, and were then transferred to the laying house at approximately 15 weeks of age. The chickens were housed individually in cages and had unrestricted access to food and water, along with regular immunization. Chickens were kept under natural light during the growing stage, followed by 16 h:8 h light:dark cycle after growing stage. The chicken coop maintained temperature control through the use of a fan humidification curtain, and feeding and manure cleaning processes were mechanized. The BW and body size were measured at 0–18 weeks. The measurement methods for some traits were as follows: Body oblique length (BOL): the distance between the shoulder joints and the sciatic tuberosity was measured along the animal’s body surface with a leather ruler. Chest breadth (CB): the distance between the two shoulder joints measured on the body surface with a caliper. Chest depth (CD): the distance from the first thoracic vertebra to the anterior edge of the keel was measured with a caliper on the body surface. Thoracic horn (TH): the angle of the thorax on both sides was measured with a thoracic corrector at the anterior edge of the keel. Keel length (KL): the distance from the anterior end of the keel eminence to the end of the keel was measured on the body surface with a caliper. Shank length (SL): the straight line distance from the upper tibial joint to the third and fourth toes measured with a caliper. Shank circumference (SC): circumference of the middle of the tibia. Pelvic width (PW): distance between the two sciatic tuberosities measured with calipers. All the phenotypic data were distributed within the range of the mean ± 3 standard deviations and passed quality control for subsequent GWAS analysis.

### Genetic materials, DNA extraction and sequencing

We collected blood samples from the wing vein of 596 chickens (supplementary Table S1) and extracted genomic DNA using the phenol-chloroform method. The DNA quality was assessed by agarose gel electrophoresis, and paired-end (2 × 150 bp) DNA libraries were constructed for each sample. The DNBSEQ sequencing platform (BGI Genomics, Shenzhen, China) was used to obtain sequence data for all libraries. Notably, the sequencing data of three chicken breeds (*n* = 50), including those from our previous research on local chicken breeds (Recessive White (RW), WB, and Luxi Mini (LM) chickens) were also used in this study. RW broilers are classified as a specialized line for meat production, known for their large body size and well-developed pectoral muscles. WB chickens, on the other hand, are versatile and utilized for both meat and egg production, characterized by medium-sized bodies. The LM chicken is a small ornamental breed that originates from China, known for its compact size. At 5 months of age, adult hens of this breed typically weigh around 0.86 kg, while adult cocks weigh approximately 1.2 kg. The accession number for these data is CRA006685 in the GSA database [19]. This study analyzed a total of 646 chickens from three breeds.

### Variant calling, quality control

Sequencing raw data was filtered with SOAPnuke (v1.5.6) [20] by removing reads containing sequencing adapter; removing low-quality data with read quality value < 20; remove reads whose unknown base (N base) ratio > 10%, and remove the reads with low quality accounting for more than 50%. The Burrows–Wheeler aligner (BWA) software [21] was used to align clean data to the chicken reference genome (http://ftp.ensembl.org/pub/release-106/fasta/gallus_gallus/), and the Samtools software [22] was used to sort the aligned sequences according to the coordinates on the genome. The Qualimap 2 tool [23] was used to obtain summary statistics to assess the effectiveness of read mapping and alignment quality, and Samtools software was used to filter out the reads with quality values less than 30. Single nucleotide polymorphisms (SNPs) were called using the GATK HaplotypeCaller v3.3 [24] with the SNP filtering conditions based on the following: Quality by Depth (QD) < 2.0, Fisher Strand (FS) > 60.0, root mean square of Mapping Quality (MQ) < 40.0, MQRankSum < -12.5, HaplotypeScore > 13.0, and ReadPosRankSum < -8.0. The SNPs obtained by preliminary filtration were selected for subsequent analysis according to the following quality control standards: SNPs with minor allele frequency (MAF) > 0.05 and missing rate < 0.1. A total of 9,406,362 biallelic SNPs were retained for subsequent analysis.

### Population genetics analysis

Principal component analysis (PCA) was performed using the software PLINK v1.9. The population structure of different admixture proportions was evaluated using the program ADMIXTURE v1.3. Three solutions (2 < k < 4) were selected for genetic clustering, and the software FigTree v1.4.0 (tree.bio.ed.ac.uk/software/figtree/) was used to visualize the phylogenetic trees.

### Analysis of nucleotide diversity, linkage disequilibrium (LD) decay

To further evaluate the genetic characteristics among different species, we determined the genetic diversity by measuring the fixation-index (Fst) using VCFtools v0.1.13 [25]. The linkage disequilibrium (LD) decay level was calculated and plotted using the PopLDdecay software [26], with a maximum distance of 500 kb.

### Detection of selective sweeps

We detected candidate divergent regions (CDRs) by searching the genome for regions with high Fst (top 1%) values. First, we calculated the Fst value along the autosomes in sliding 40-kb windows with 10-kb steps using VCFtools software and in-house scripts, by comparing values among WB, RW and LM chickens. We restricted our CDR descriptions to the top 1% most significant windows in Fst values, as these windows represented the extreme ends of the distributions.

### Estimation of genetic parameters

SNP-based heritability (h^2^ SNP) was calculated using the GCTA v1.93.2 beta software [27] based on the genetic relationship matrix (GRM) between pairs of individuals [28]. The restricted maximum likelihood (REML) method was used for genetic parameter estimation. The genetic-statistical model was defined as follows:

$$ {Y}_{i}={X}_{i}{b}_{i}+{Z}_{i}{u}_{i}+{e}_{i}$$

where $$ {Y}_{i}$$ is a vector of clutch traits; $$ {X}_{i}$$ and $$ {Z}_{i}$$ are incidence matrices for $$ {b}_{i}$$ and $$ {u}_{i}, $$respectively; bi is a vector of fixed effect; $$ {u}_{i}$$ is a vector of polygenic effects with a variance-covariance structure of $$ u{\sim} N\left(0,G{\sigma }_{u}^{2}\right);$$ G is the GRM between individuals; $$ {\sigma }_{u}^{2} $$is the polygenic variance; $$ {e}_{i}$$ is a vector of random residual effects with $$ {e}_{i}{\sim}N(0,I{\sigma }_{e}^{2})$$; I is an identity matrix of dimension n × n (with a sample size *n*= 596).

### Genome-wide association study for body weight and body size in Wenshang Barred chicken

WB chickens were selected based on meat production traits over multiple generations, and SNP information and phenotypic records were comprehensively collected. To investigate the genetic basis of BW and body size, association analysis of BW, chest breadth (CB), chest depth (CD), thoracic horn (TH), body oblique length (BOL), keel length (KL), pelvic width (PW), shank length (SL) and shank circumference (SC) was performed using the linear mixed model in the Genome-wide Efficient Mixed Model Association (GEMMA) software (v0.98.4) based on chickens genotyped by whole-genome sequencing. After quality control (-- mind 0.1, --maf 0.05) using the PLINK v1.9 software, a total of 9,406,362 SNPs were retained, and GWAS was performed as follows:

$$ y=W\alpha +\text{X}{\upbeta }+\text{u}+\text{e}$$

where y denotes the vector of phenotypic values; W represents the vector of covariates, including a column of 1 s; α is the vector of the corresponding coefficients including the intercept; x represents the vector of marker genotypes; β denotes the effect size of the marker; u represents the vector of random polygenic effects; e is the vector of errors. The Wald test was used as a criterion to select SNPs associated with metabolizable efficiency traits. Similarly, the whole-genome and suggestive significance thresholds were corrected by the Bonferroni test (0.05/9,406,362 and 0.01/9,406,362, respectively). Additionally, Manhattan and quantile-quantile (Q-Q) plots were visualized using the CMplot package in the R environment. The LD blocks of target regions were performed using the Haploview v4.2 software.

### Statistical analysis

Statistical analyses were performed using SPSS 25.0 software (IBM Corporation, Armonk, NY, USA) or R environment.

## Results

### Whole-genome sequencing and variation

Following standardized procedures for library construction and whole-genome sequencing using the BGISEQ platform, we obtained 4.15 Tb of raw data (supplementary Table S1) for 596 individuals, with a mean coverage of 7.03X (supplementary Table S1). After performing quality control, the total reads per individual were 51,113,663, with a mean mapping ratio of 99.73%. These data satisfied the requirements for subsequent analyses.

### Phylogenetic and demographic analyses

**Fig. 1:**
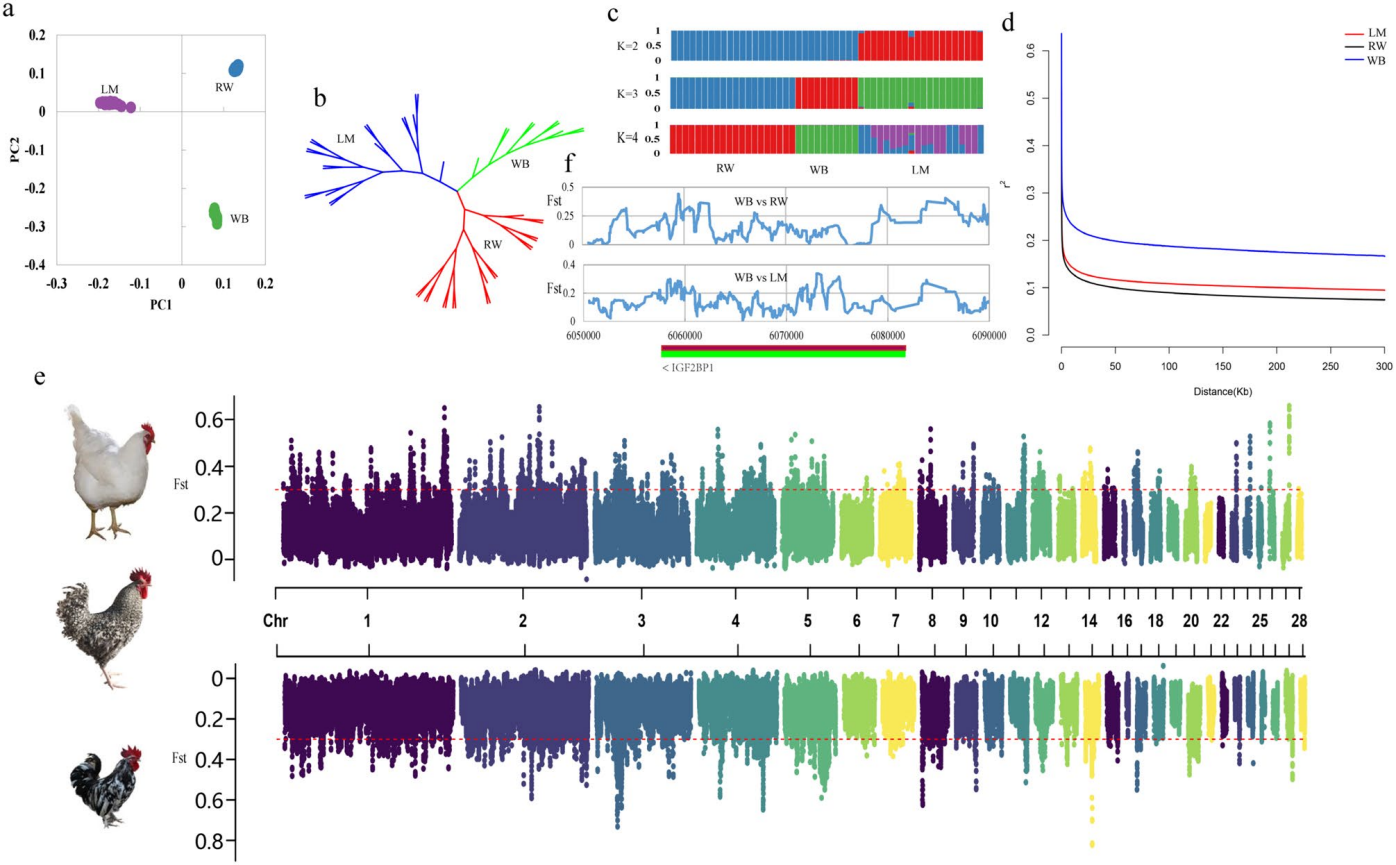
Population genetic diversity and demographic history inferences (**a**) PCA plot with three chicken breeds (**b**) Neighbor-joining tree constructed by genetic distance among three chicken breeds (**c**) Population structure analysis of three body size of chickens, where the number of ancestral clusters were set from K = 2–4. (**d**) LD decay in three body size of chickens. (**e**) Detection of selective sweep windows in purebred chickens. The red dash line indicates the top 1% threshold of Fst values. (f) Putative selected windows and genes on the chromosome 27:6.05–6.09 Mb region. RW: recessive white chickens; WB: Wenshang Barred chickens; LM: Luxi mini chickens

We conducted a comprehensive analysis of the genetic relationships among three different chicken breeds with varied body sizes. First, we used QC SNPs and performed PCA on the three breeds, which revealed a significant genetic difference among RW, WB, and LM chickens (Fig. 1a). Second, we used pairwise genetic distances to construct a neighbor-joining tree (Fig. 1b). Third, genetic coancestry analysis was performed by assuming different number of ancestral populations (K = 2–4, Fig. 1c) to classify the chickens into groups. It was found that the LD decay distance was different among the three breeds (Fig. 1d). As expected, the fast-growing RW, WB, and LM chickens were genetically distant from each other.

### Genomic signatures in purebred WB chickens

We performed the Fst test based on allele frequency differentiation with a 40 kb window size and a step size of 10 kb to identify the genomic loci that underwent selective sweeps among the three chicken breeds (Fig. 1e). By overlapping the results of the Fst analysis, we identified 287 protein-coding genes in the RW and WB populations and 241 protein-coding genes in the LM and WB populations (Fig. 1e, supplementary Table S2–3). Notably, we detected a lead signal on chromosome 5 in the RW breed, which annotated the *INS* and *IGF2* genes that play key roles in the skeletal muscle development process [29]. We also examined the *IGF2BP1* gene and found significant differences in the Fst values among the RW, WB, and LM chickens (Fig. 1f). Subsequently, we identified the list of genes harboring the top selective sweep windows (Table S4). For instance, the known growth factors *HBEGF, VEGFA, FGF23*, and *FGF6* play a crucial role in body development. Additionally, *TBX20* acts as a transcriptional activator and repressor required for cardiac development and is responsible for maintaining functional and structural phenotypes in adult heart. *TOLLIP* is a Toll-interacting protein and an essential component of the signaling pathway of IL1B and Toll-like receptors. Also, *TBX5* is involved in heart development and limb pattern formation. In addition, we also conducted KEGG pathway and GO term enrichment analyses on the gene sets from the 453 selective sweep genes in all chickens (Table S5 and S6, Fig. S1). Based on the phenotype or physiological process, the GO terms were classified into several clusters, including autophagy (e.g., GO:0006995, GO:0006914 and GO:0016236), energy metabolism (e.g., GO:0009060, GO:0022900, GO:0006091, GO:0007005, GO:0055114), and growth (e.g., GO:0071363, GO:0007169, GO:0007167).

### Descriptive statistics of traits

We calculated the descriptive statistics for the traits related to BW and body size (Table 1). The coefficients of variation of these traits in the population ranged from 4.62 to 12.94%. The SNP-based heritability estimates for the BW traits (0.47 - 0.67) and shank traits (0.33 - 0.59) were high, but they were relatively low (0.04 - 0.05) for TH traits, breast muscle traits, and body size traits.

**Table 1:** Descriptive statistics for BW and body size traits of WB chicken

Trait*	N	Mean	SD	MAX	Min	C.V	h2
BW (0wk)	596	34.33	3.27	59.72	23.74	9.54%	0.67
BW (2wk)	596	106.97	11.74	145.00	69.00	10.98%	0.59
BW (4wk)	596	231.64	27.32	320.00	137.00	11.79%	0.47
BW (6wk)	596	399.25	47.90	555.00	216.00	12.00%	0.47
BW (8wk)	596	552.87	67.58	848.00	314.00	12.22%	0.47
BW (10wk)	596	701.56	90.79	1035.00	404.00	12.94%	0.60
BW (12wk)	596	887.89	104.86	1312.00	544.00	11.81%	0.62
BW (14wk)	596	1032.12	121.14	1475.00	618.00	11.74%	0.51
BW (16wk)	596	1140.40	131.83	1640.00	670.00	11.56%	0.51
BW (18wk)	596	1264.13	150.60	1833.00	752.00	11.91%	0.64
CB (8wk)	596	40.87	2.95	51.57	30.09	7.22%	0.37
CB (18wk)	596	60.00	3.62	70.80	47.16	6.03%	0.50
CD (8wk)	596	74.91	4.44	87.96	59.72	5.93%	0.28
CD (18wk)	596	94.60	5.47	113.13	59.25	5.78%	0.27
TH (8wk)	596	58.66	3.03	67.50	49.00	5.16%	0.05
TH (18wk)	596	61.14	3.41	69.80	51.70	5.58%	0.04
BOL (8wk)	596	13.93	0.73	16.20	10.70	5.21%	0.21
BOL (18wk)	596	18.69	1.34	23.10	15.80	7.16%	0.22
KL (8wk)	596	7.48	0.52	9.20	6.00	6.96%	0.23
KL (18wk)	596	10.55	0.82	13.20	7.50	7.76%	0.45
PW (8wk)	596	33.28	2.66	62.51	26.19	8.00%	0.29
PW (18wk)	596	51.00	3.30	74.62	40.49	6.46%	0.38
SL (8wk)	596	61.32	3.65	73.85	45.79	5.96%	0.43
SL (18wk)	596	74.82	3.80	89.81	61.97	5.07%	0.59
SC (8wk)	596	3.19	0.15	3.60	2.80	4.62%	0.56
SC (18wk)	596	3.63	0.19	4.60	3.20	5.36%	0.33

* BW: body weight; CB: chest breadth; CD: chest depth; TH: thoracic horn; BOL: body oblique length; KL: keel length; PW: pelvic width; SL: shank length; SC: shank circumference

### GWAS and fine-mapping for body weight and body size traits

**Fig. 2:**
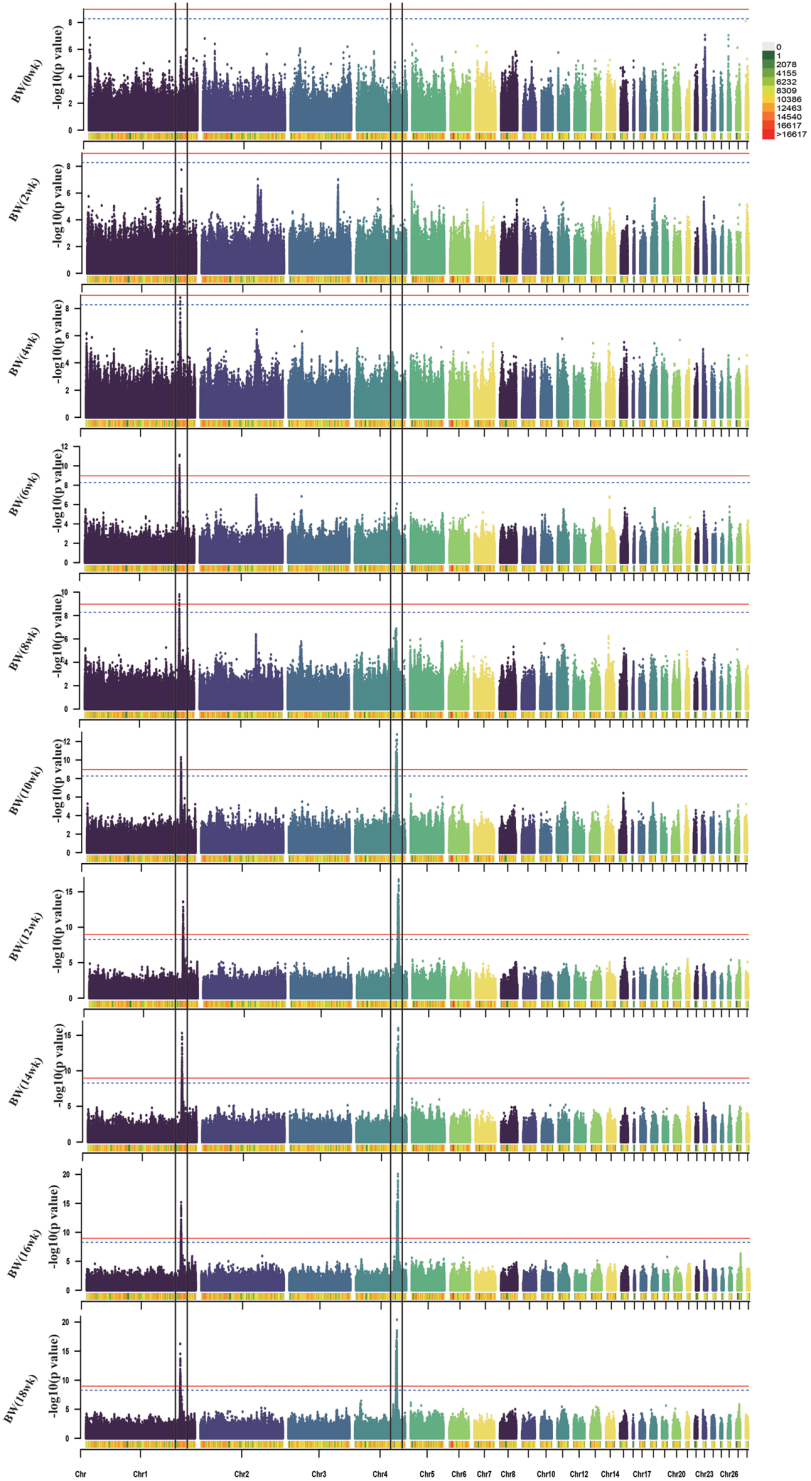
Manhattan plots of GWAS for BW traits in WB chicken. Each dot represents a SNP in the dataset. The horizontal red and blue lines indicate the thresholds for genome-wide significance (P value = 1.07e-09) and suggestive significance (P value = 5.32e-09), respectively. BW: body weight

**Fig. 3:**
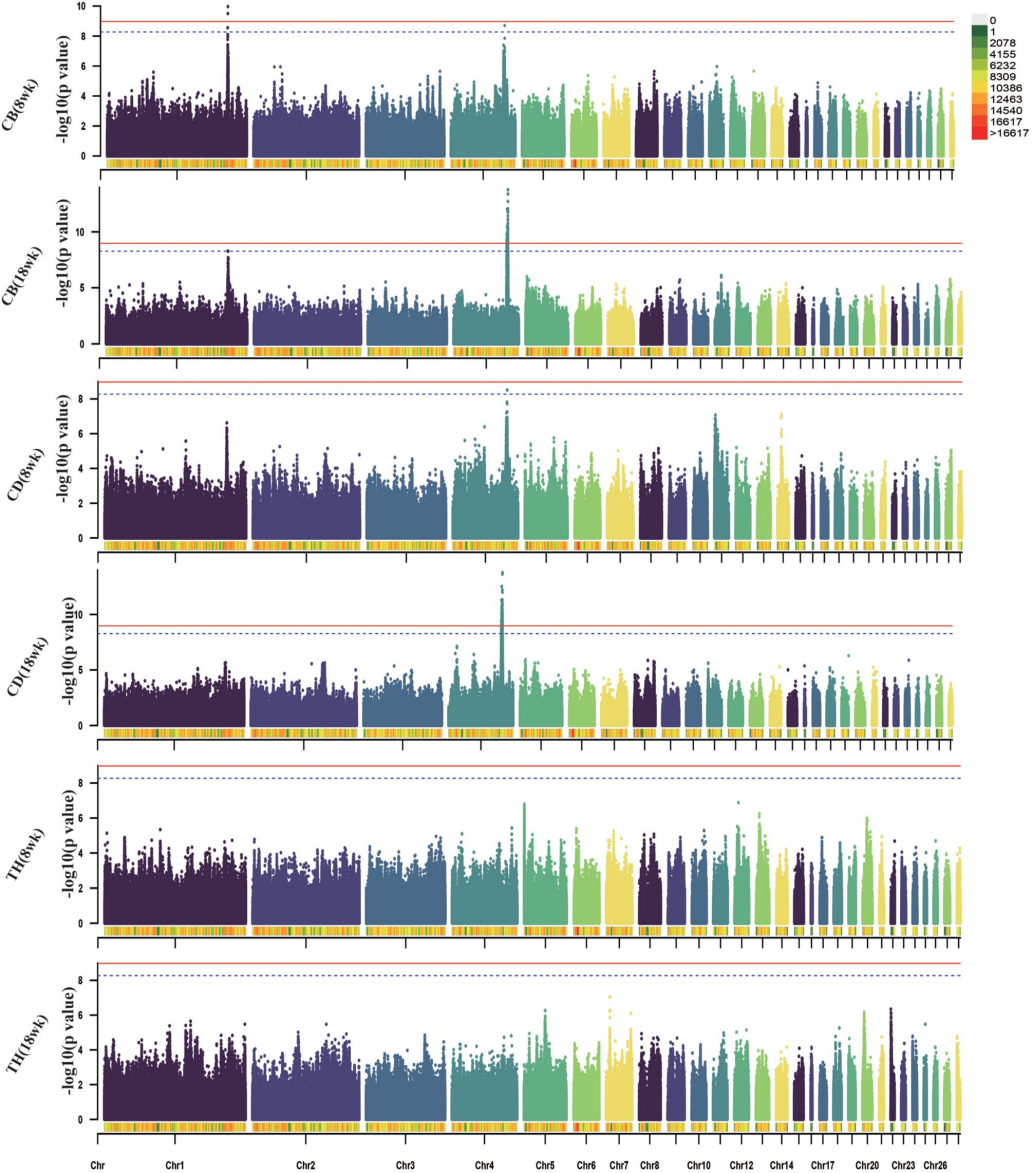
Manhattan plots of GWAS for breast muscle size traits in WB chicken. Each dot represents a SNP in the dataset. The horizontal red and blue lines indicate the thresholds for genome-wide significance (P value = 1.07e-09) and suggestive significance (P value = 5.32e-09), respectively. CB: chest breadth; CD: chest depth; TH: thoracic horn

**Fig. 4:**
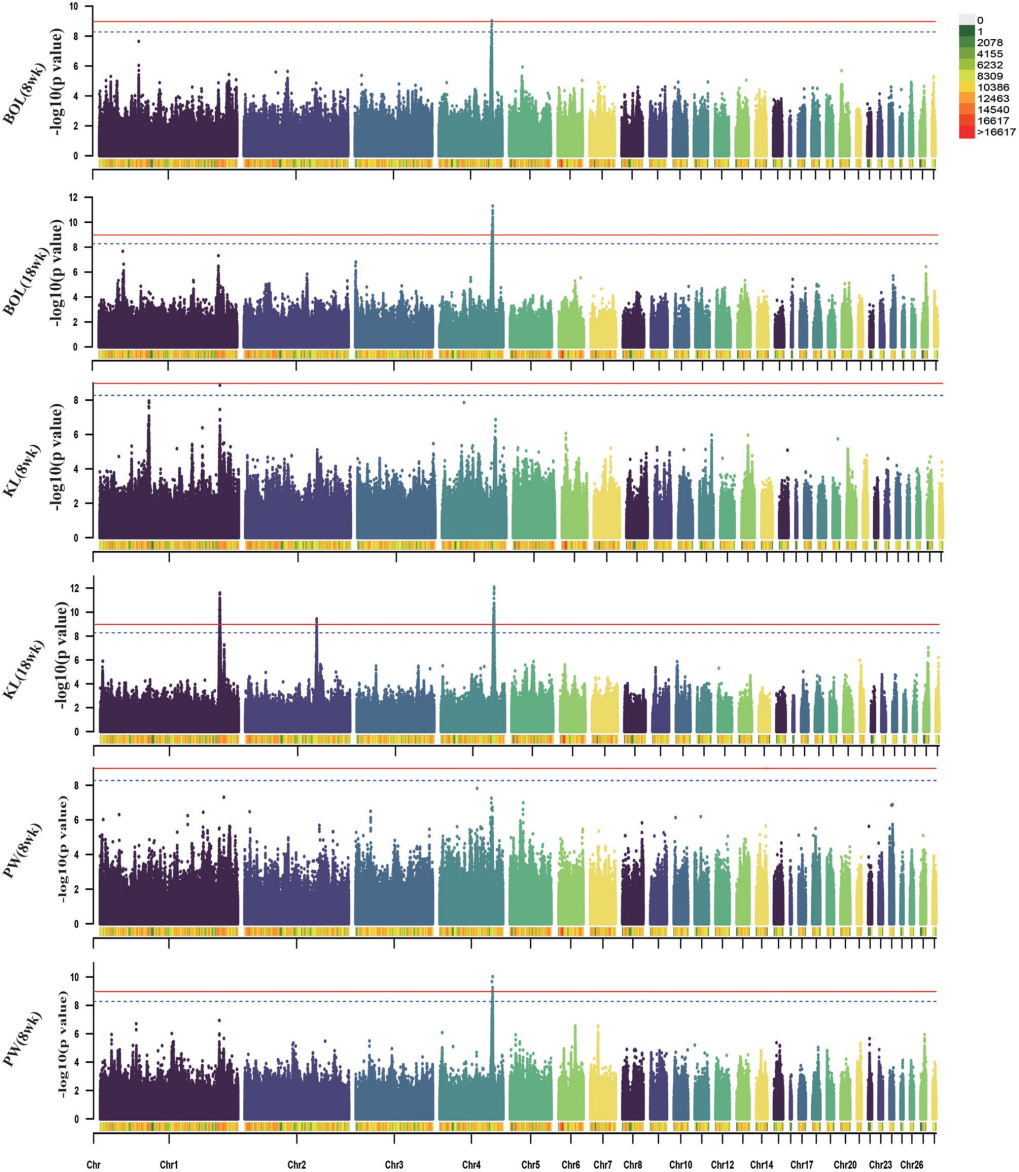
Manhattan plots of GWAS for body size traits in WB chicken. Each dot represents a SNP in the dataset. The horizontal red and blue lines indicate the thresholds for genome-wide significance (P value = 1.07e-09) and suggestive significance (P value = 5.32e-09), respectively. BOL: body oblique length; KL: keel length; PW: pelvic width

**Fig. 5:**
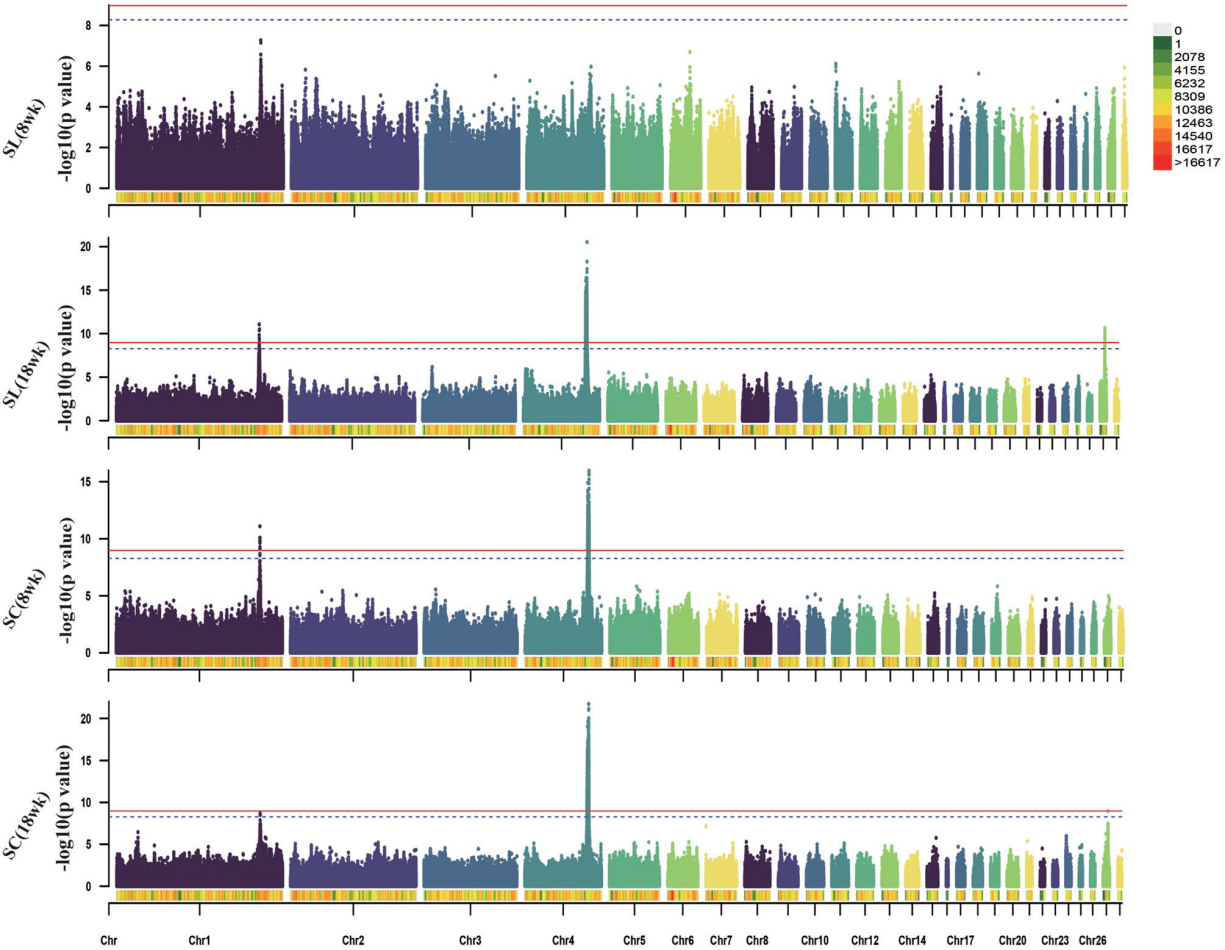
Manhattan plots of GWAS for SL and SC traits in WB chicken. Each dot represents a SNP in the dataset. The horizontal red and blue lines indicate the thresholds for genome-wide significance (P value = 1.07e-09) and suggestive significance (P value = 5.32e-09), respectively. SL: shank length; SC: shank circumference

This study mainly focused on analyzing BW and body size traits. The Manhattan plots and significant SNPs are shown in Figs. 2, 3, 4 and 5, and the corresponding Tables 2, 3, 4 and 5. The Q-Q plots are shown in Fig. S3, S3, S4, and S4, and Table S7 shows the significant SNPs for all phenotypes. The additive effects of lead SNPs estimated by GEMMA are shown in Table S8.

We identified two significant regions, one on chromosome 1 (170.4-171.5 Mb) and the other on chromosome 4 (74.1–76.4 Mb), for the ten BW traits. The chromosome 1 region was found to be correlated with BW during the entire growth stage. In particular, the largest region associated with BW at 16 weeks contained 218 significant SNPs, implicating genes such as *RB1*, *RCBTB2*, *CAB39L*, *SETDB2*, *PHF11*, *ARL11, KPNA3, SPRYD7, RNASEH2B*, and *WDFY2*. In contrast, the chromosome 4 region was associated with BW traits after 10 weeks of age. The most significant interval was correlated with BW at 16 weeks of age, comprising 1,076 significant SNPs, involving genes such as *PPARGC1A, KCNIP4, SLIT2, LCORL, LDB2*, and *LAP3*.

**Table 2:** Overview of the significant SNPs associated with BW traits in WB chicken

Trait*	chr	Base-pair region		nSNP	Related genes
		Start	End		
BW (4wk)	1	170,467,569	170,781,981	4	*CAB39L*
BW (6wk)	1	170,467,569	170,982,124	25	*CAB39L, SETDB2, PHF11, KPNA3, SPRYD7*
BW (8wk)	1	170,666,389	170,878,448	11	*/*
BW (10wk)	1	170,563,091	171,531,433	23	*PHF11, WDFY2*
	4	74,106,930	76,254,700	138	*PPARGC1A, KCNIP4, SLIT2, LCORL, LDB2*,
BW (12wk)	1	170,462,467	171,531,433	104	*CAB39L, PHF11, SETDB2, SPRYD7, WDFY2*
	4	74,104,662	76,338,507	766	*PPARGC1A, KCNIP4, SLIT2, LCORL, LDB2, LAP3*
BW (14wk)	1	170,144,714	171,531,433	180	*RB1, RCBTB2, CAB39L, SETDB2, PHF11, ARL11, KPNA3, SPRYD7, RNASEH2B, WDFY2*
	4	74,106,696	76,254,700	518	*PPARGC1A, KCNIP4, SLIT2, LCORL, LDB2, LAP3*
BW (16wk)	1	170,144,454	171,531,433	218	*RB1, RCBTB2, CAB39L, SETDB2, PHF11, ARL11, KPNA3, SPRYD7, RNASEH2B, WDFY2*
	4	74,106,686	76,419,083	1076	*PPARGC1A, KCNIP4, SLIT2, LCORL, LDB2, LAP3*
BW (18wk)	1	170,144,714	171,531,433	172	*RB1, RCBTB2, CAB39L, SETDB2, PHF11, ARL11, KPNA3, SPRYD7, RNASEH2B, WDFY2*
	4	74,104,662	76,338,507	1073	*PPARGC1A, KCNIP4, SLIT2, LCORL, LDB2, LAP3*

*BW: body weight

We also identified breast muscle size traits on chromosome 4 (74.4–76.2 Mb). Specifically, 167 significant SNPs were associated with the CB trait, and 343 significant SNPs were correlated with the CD trait, involving the following genes: *SLIT2, KCNIP4, LAP3, LCORL*, and *LDB2.*

**Table 3:** Overview of the significant SNPs associated with breast muscle size traits in WB chicken

Trait*	chr	Base-pair region		nSNP	Related genes
		Start	End		
CB (8wk)	1	170,529,013	170,781,981	3	*/*
	4	76,071,453		1	*LDB2*
CB (18wk)	1	170,563,091		1	*PHF11*
	4	74,466,678	76,178,650	167	*SLIT2, KCNIP4, LAP3, LCORL, LDB2*
CD (8wk)	4	76,071,453		1	*LDB2*
CD (18wk)	4	74,497,667	76,243,602	343	*SLIT2, KCNIP4, LAP3, LCORL, LDB2*

*CB: chest breadth; CD: chest depth

For body size traits, 99 significant SNPs found to be associated with the 18-week BOL trait were located on the *SLIT2* and *LCORL* genes. The 167, 19, and 175 significant SNPs located on chromosomes 1, 2 and 4, respectively, were associated with the 18-week KL trait, and involved the genes *ITM2B, CAB39L, SETDB2, PHF11, ARL11, KPNA3, SPRYD7, YES1, COLEC12, SLIT2, LCORL*, and *LDB2*. A total of 24 significant SNPS located in the 75.07 -76.24 Mb region of chromosome 4 were correlated with PW traits, and the annotated genes included *SLIT2, LCORL* and *LDB2.*

**Table 4:** Overview of the significant SNPs associated with body size traits in WB chicken

Trait*	chr	Base-pair region		nSNP	Related genes
		Start	End		
BOL (8wk)	4	75,813,782	75,814,416	2	*/*
	4	76,071,222	76,071,453	2	*LDB2*
BOL (18wk)	4	75,069,271	76,170,166	99	*SLIT2, LCORL*
KL (8wk)	1	170,762,645		1	*/*
KL (18wk)	1	169,943,496	171,292,565	167	*ITM2B, CAB39L, SETDB2, PHF11, ARL11, KPNA3, SPRYD7*
	2	101,935,680	102,103,490	19	*YES1, COLEC12*
	4	74,501,614	76,338,507	175	*SLIT2, LCORL, LDB2*
PW (8wk)	14	14,011,969		1	*/*
PW (18wk)	4	75,069,271	76,244,364	24	*SLIT2, LCORL, LDB2*

*BOL: body oblique length; KL: keel length; PW: pelvic width

For the SL traits, 1,505 significant SNPs were located on chromosomes 1, 4 and 27. The 21 significant SNPs were clustered within a 48.25-kb region (chromosome 1:170.56–171.04 Mb), 1,458 SNPs were clustered within a 2.31-Mb region (chromosome 4:74.1–76.1 Mb) and 26 SNPs were clustered within a 191-kb region (chromosome 27:5.95–6.14 Mb). The genes *PHF11, SPRYD7, PPARGC1A, SLIT2, KCNIP4, LCORL, LAP3, QDPR, LDB2, IGF2BP1*, and *GIP* were annotated in the above significant regions. For the SC traits at 8 weeks and 18 weeks, 780 and 1,931 significant SNPs, respectively, were located in the 73.87-76.45 Mb region of chromosome 4 with a total of 2.57 Mb, involving the genes *PPARGC1A, KCNIP4, SLIT2, LCORL, LAP3, LDB2*, and *TAPT1.*

**Table 5:** Overview of the significant SNPs associated with SL and SC traits in WB chicken

Trait*	chr	Base-pair region		nSNP	Related genes
		Start	End		
SL (18wk)	1	170,560,919	171,043,404	21	*PHF11, SPRYD7*
	27	5,952,667	6,144,035	26	*IGF2BP1, GIP*
	4	74,106,686	76,419,083	1,458	*PPARGC1A, SLIT2, KCNIP4, LCORL, LAP3, QDPR, LDB2*
SC (8wk)	1	170,666,389	170,832,813	10	*/*
	4	74,067,581	76,338,507	780	*PPARGC1A, KCNIP4, SLIT2, LCORL, LAP3, LDB2*
SC (18wk)	1	170,832,813	170,917,528	2	*/*
	4	73,876,680	76,454,847	1,931	*PPARGC1A, KCNIP4, SLIT2, LCORL, LAP3, LDB2, TAPT1*
	27	5,939,929		1	*MEOX1*

*SL: shank length; SC: shank circumference

### The *LCORL*, *LDB2* and *PPARGC1A* gene is a potential causal gene for body weight and body size

**Fig. 6:**
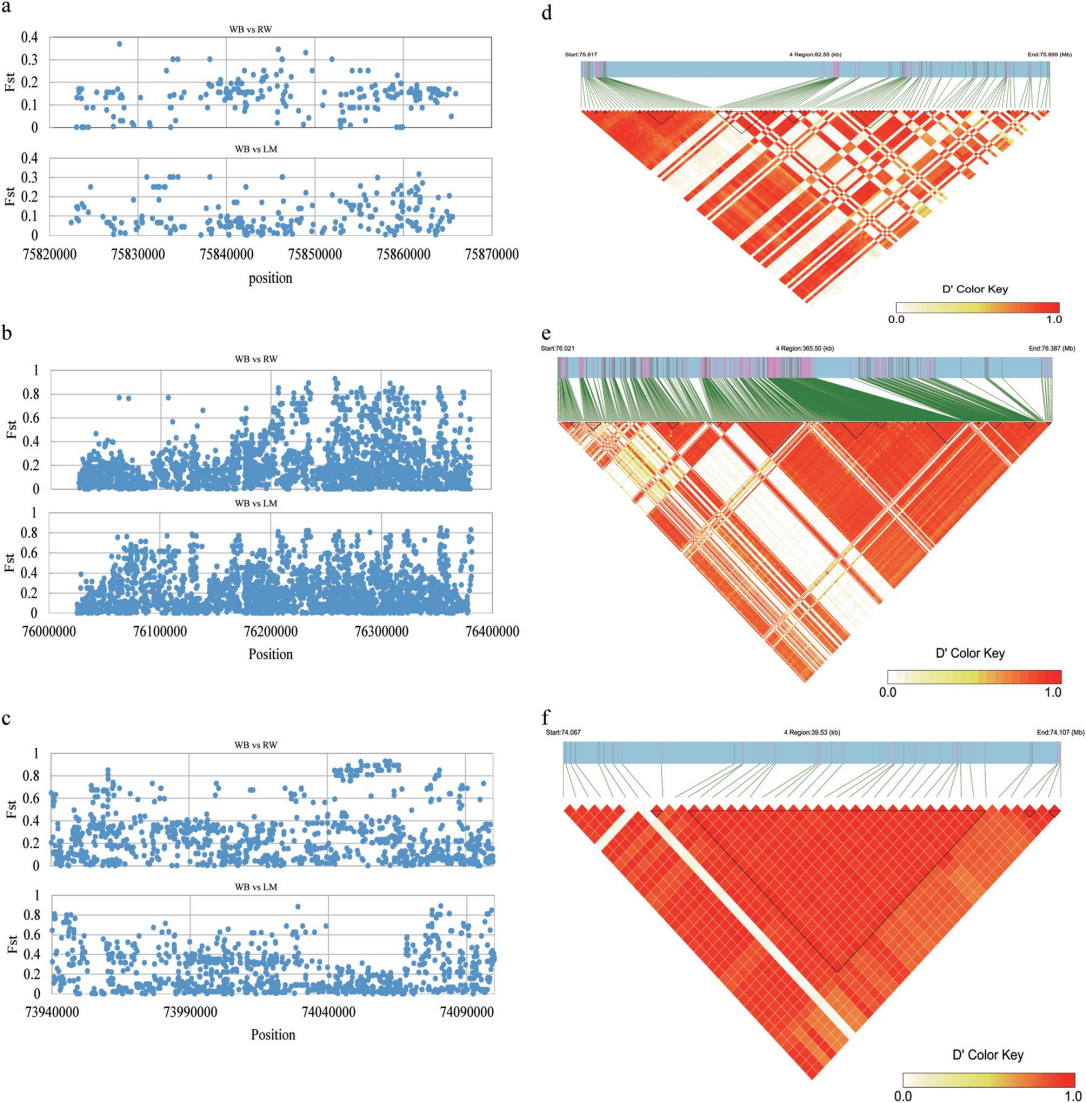
Association results of the candidate region on chromosome 4 for BW and body size traits. (**a**, **b**, **c**) Putative selected SNPs in the *LCORL*, *LDB2* and *PPARGC1A* genes. RW: recessive white chickens; WB: Wenshang Barred chickens; LM: Luxi mini chickens. (**d**, **e**, and **f**) Linkage disequilibrium (LD) analysis of the overlap significant SNPs on the *LCORL*, *LDB2* and *PPARGC1A* genes

The *LCORL, LDB2*, and *PPARGC1A* genes were significantly correlated with 13, 12, and 8 traits, respectively, based on the results of the above analysis. In this section, we focus on these three genes and analyze their polymorphism in the three chicken breeds (RW, WB, and LM) with varied body sizes. The Fst analysis results indicated that the significant SNPs present on these genes showed apparent differences among breeds with pronounced differences in BW and body size (Fig. 6a, b and c). Furthermore, we observed that the related SNPs showed a strong linkage in the WB chicken breed (Fig. 6d, e and f). These findings suggest the possibility that the genome regions of these three genes might have undergone natural selection during the development of different chicken breeds.

## Discussion

The domestic chicken is an ideal model to investigate the genetics of phenotypic evolution [30]. Evolving poultry genetics and breeding have led to a diverse range of phenotypes and demographic history in local breeds [31, 32]. Domestication has also limited phenotypic differences among local breeds by selecting for genetic variants that favor traits leading to improved production [33]. Among these traits, animal body size plays a critical role in the profitability of poultry meat. Therefore, optimizing this trait has been an important goal during domestication [33, 34]. Selection for particular traits is the decisive factor behind the substantial rise in productivity, accounting for more than 90% of the improvement [35]. We conducted a comprehensive genetic diversity study and selective sweep analysis in fast-growing (RW chickens), local chicken (WB chicken), and mini chicken (LM chicken). The selective sweep analysis identified 455 protein-coding genes that underwent positive selection and played a critical role in domestication and breeding processes for phenotypes associated with muscle growth, reproduction, brain development, growth factors, and disease resistance. The significant GO enrichment terms were related to the insulin and GnRH signaling pathways in chickens.

Chicken breeds exhibit great variation in size in response to natural and/or artificial selection [36]. Understanding the genetic mechanisms underlying this variability in chicken body size is still inadequate. The chicken body size primarily reflects the growth of muscles and bones [37, 38], making growth a crucial selection criterion in chicken breeding. Genetically, chicken body size is a complex trait influenced by several genes on autosomal and sex chromosomes. Hundreds of QTLs have been mapped on autosomes for body size-related traits, such as SL, KL, and BW [37, 39–44].

A genome-wide association study was conducted to analyze the body size of Asian pheasants and Asian bantams. The study found a region on chromosome 4 (GGA4:17.3–21.3 Mb) that contained a total of 60 genes. Two notable genes in this region are myotubularin 1 (*MTM1*) and secreted frizzled-related protein 2 (*SFRP2*), both of which are potential candidate genes associated with body size traits [44]. The previous GWAS study included 541 chickens from 23 regional breeds in Italy, with each breed consisting of 20 to 24 chickens. Significant SNPs were found in the genome-wide association study, specifically associated with dwarfism in the dwarf breeds. These breeds shared a candidate genomic region on chromosome 1, where significant SNPs were found within the *LEMD3* and *HMGA2* genes [45]. This study examined the genome-wide association of 10 BW traits and 16 body size traits. We confirmed a total of 2,522 genome-wide significant SNPs, most of which were present on chromosomes 1 and 4. The 72 ${\sim}$ 76 Mb region of chromosome 4 contained genes, such as *PPARGC1A, KCNIP4, SLIT2, LCORL, LDB2*, and *LAP3*. The GWAS of F2 progenies showed that the *LDB2* gene was associated with BW at 7 ${\sim}$ 12 weeks and average daily gain at 6 ${\sim}$ 12 weeks [17]. A previous GWAS study was conducted using the chicken 60 K SNP panel on 1,328 Korean native chickens to analyze body weight (BW) traits.The results identified twelve single nucleotide polymorphisms (SNPs) associated with BW at the suggestive significance level.These SNPs were found near or within 11 candidate genes, specifically *WDR37*, *KCNIP4*, *SLIT2*, *PPARGC1A*, *MYOCD*, and *ADGRA3* [46]. Some of the genes overlapped with the results of this study. The *NCAPG-LCORL* locus is widely believed to impact human height in human studies. In the GWAS of cattle and horses and the whole-genome selective sweep analysis of pigs and dogs, the *NCAPG-LCORL* locus was found to be significantly associated with body length and BW [47]. Furthermore, *PPARGC1A* has been shown to facilitate mitochondrial biogenesis and modulate skeletal muscle metabolism by mediating the flux of glycolysis and the tricarboxylic acid (TCA) cycle, which drives the transformation of fast-twitch myofibers to slow-twitch myofibers, thus increasing chicken skeletal muscle mass [48]. In another region, the *CAB39L* and *WDFY2* genes were identified as candidates on chromosome 1 (170.5-171.5 Mb) and associated with BW from 4 to 18 weeks of age and body size traits. The *CAB39L* gene can be considered a novel candidate gene for chicken growth and development [49]. The *WDFY2* may be a candidate susceptibility gene located downstream of *TP63* in the network of limb development [50]. Other genes, such as *IGF2BP1* and *GIP*, were found to be associated with the SL trait. The *GIP* gene encodes an incretin hormone that induces insulin secretion [51] and mediates appetite and energy intake [52].

In domestic animals, loci with a significant positive effect on favorable traits tend to undergo strong selection and fixation. In this study, we investigated the genomic variations of the *LCORL, LDB2*, and *PPARGC1A* genes in the chromosome 4:72 ${\sim}$ 76 Mb region by performing selective sweep analysis. The results indicated that these genes not only were associated with BW and body size traits, as revealed by the GWAS, but also were strongly selected and fixed among breeds with differences in BW and size. Thus, it is likely that the chromosome 4:72 ${\sim}$ 76 Mb region harbors loci that significantly impact BW and body size.

Observations of the same individual at multiple time points are called longitudinal traits and provide a better representation of growth and production in farm animals than single data records [53–57]. The BW of chickens at different weeks of age is a classic example of a longitudinal trait. In this study, we performed GWAS independently for each time point to identify the genetic basis of BW. However, a more effective strategy would be to fit the growth curve and use the fitted parameters to conduct the association analysis. This approach would better reflect the growth trajectory and provide a novel insight into the genetic underpinnings of BW in chickens.

## Conclusion

In conclusion, our GWAS identified 2,522 SNPs with genome-wide significance, the majority of which are being reported for the first time. Several SNP effects overlapped with previously reported QTL regions, supporting the validation of QTL effects. Using a combination of GWAS and FST-based approaches, we identified three genes (*LCORL, LDB2*, and *PPARGC1A*) in the Chinese WB chicken associated with BW and body size traits. Our study provides important insights into the evolution and genetic basis of Chinese local chickens, which may be beneficial for both domestic and international chicken breeders. This study may also contribute to the development of genome-scale selective breeding strategies aimed at increasing chicken meat yield.

### Electronic supplementary material

Below is the link to the electronic supplementary material.

Supplementary Material 1

Supplementary Material 2

## Data Availability

The raw sequence data reported in this paper have been deposited in the Genome Sequence Archive in National Genomics Data Center, China National Center for Bioinformation / Beijing Institute of Genomics, Chinese Academy of Sciences (GSA: CRA011183) that are publicly accessible at https://ngdc.cncb.ac.cn/gsa.

## Abbreviations

BOL: body oblique length
BW: body weight
CB: chest breadth
CD: chest depth
CDRs: candidate divergent regions
Fst: fixation-index
GRM: genetic relationship matrix
GWAS: genome-wide association study
KL: keel length
LD: linkage disequilibrium
LM: luxi Mini chickens
MAF: minor allele frequency
PCA: principal component analysis
PW: pelvic width
QTLs: quantitative trait loci
REML: restricted maximum likelihood
RW: recessive White chickens
SC: shank circumference
SL: shank length
SNPs: single nucleotide polymorphisms
TH: thoracic horn
WB: wenshang Barred chickens
