# Supplementary material for: Elucidation of the genetic determination of body weight and size in Chinese local chicken breeds by large-scale genomic analyses
Source: BMC Genomics. 2024 Mar 20;25:296. doi: 10.1186/s12864-024-10185-6 (PMC10956266; doi:10.1186/s12864-024-10185-6)
Supplement: Supplementary file 1 — Supplementary Material 1 [file 12864_2024_10185_MOESM1_ESM.docx]

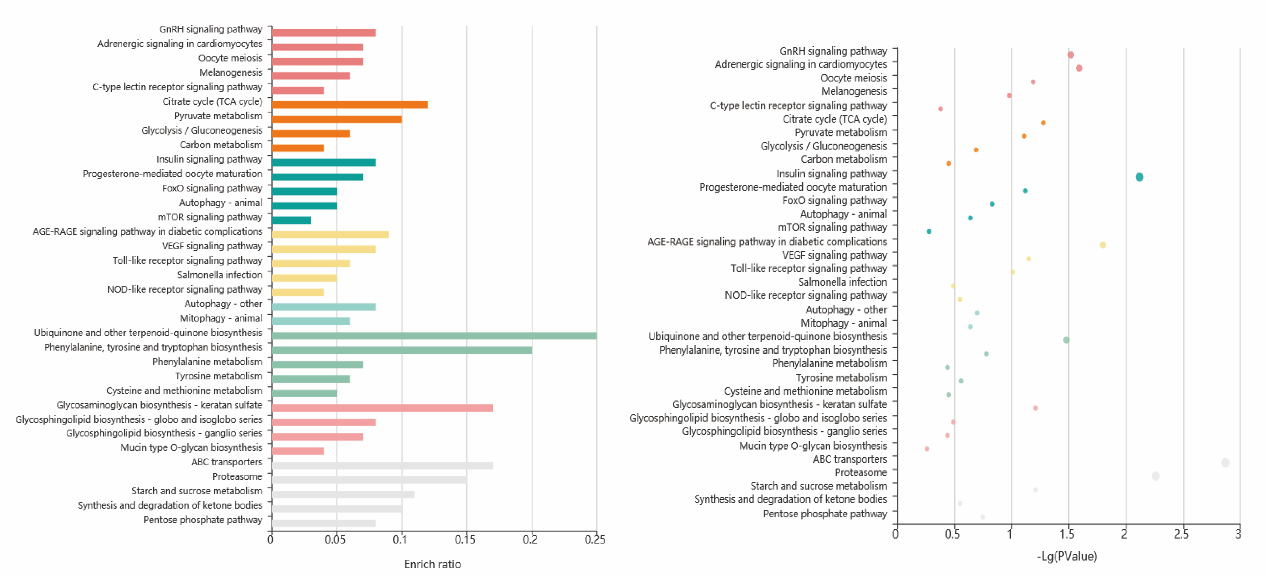


Fig.S1 GO enrichment results based on genes carrying significant sweep signals.


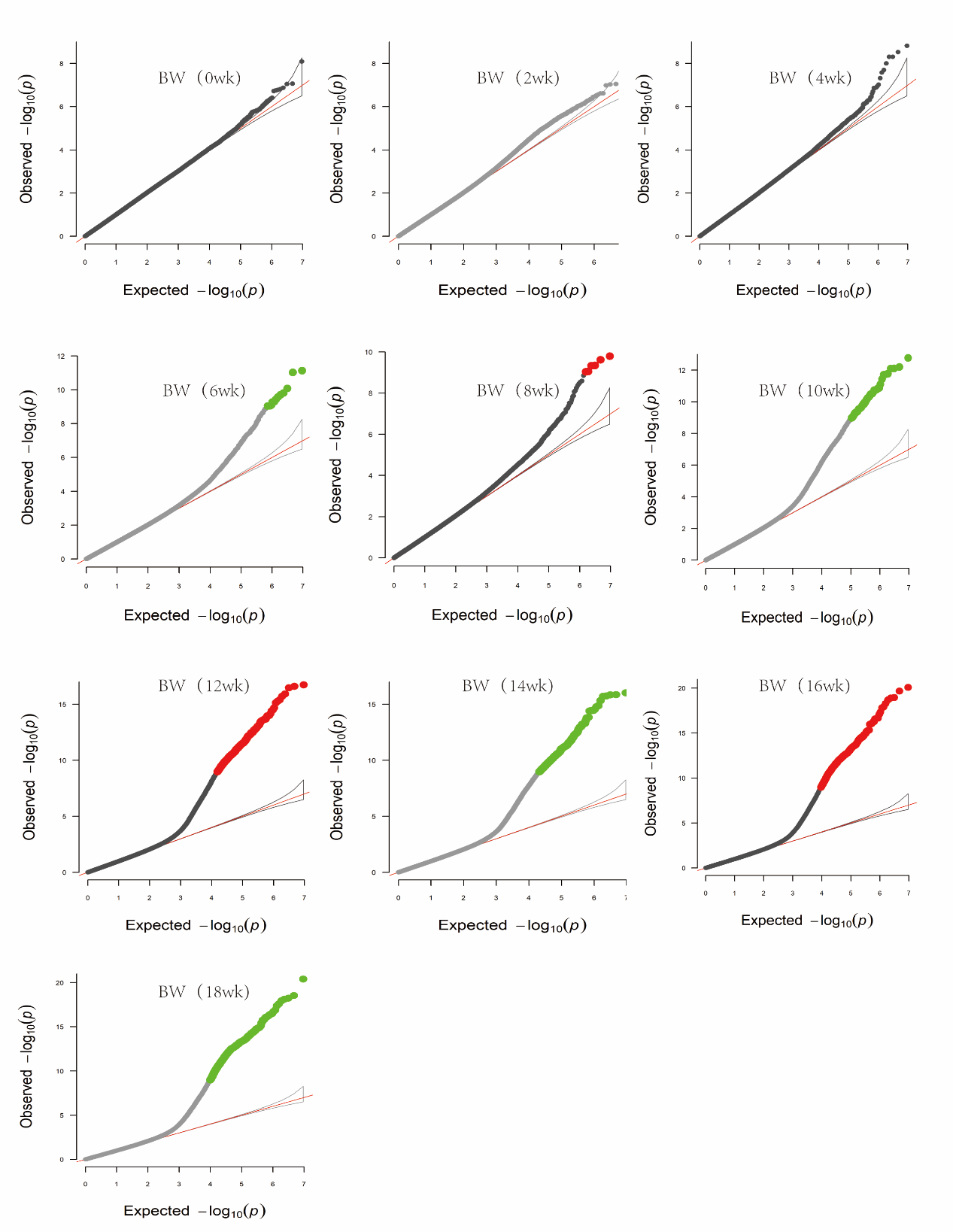


Fig.S2 QQ plots of GWAS for body weight traits in WB chicken. BW body weight.


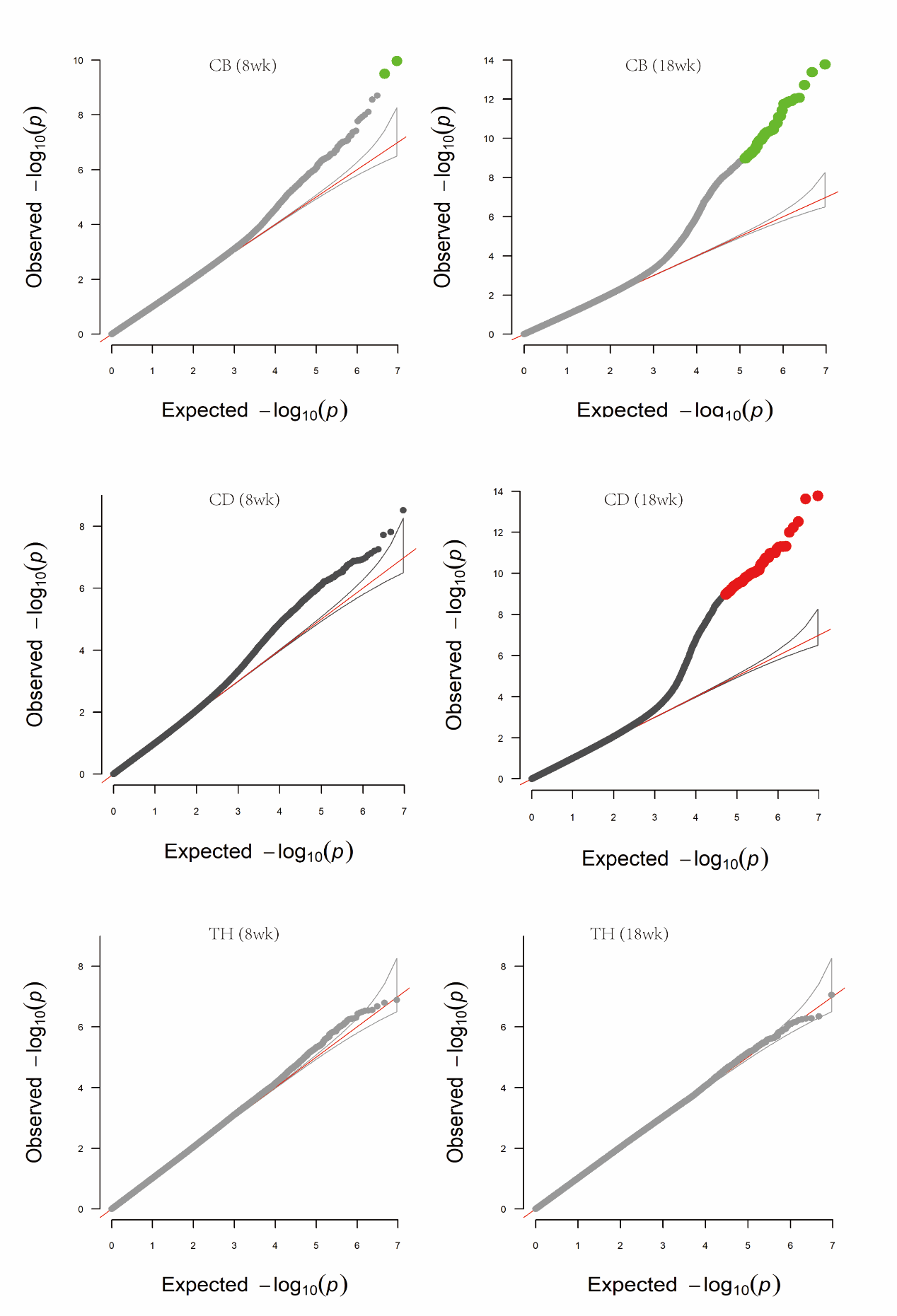


Fig.S3 QQ plots of GWAS for breast muscle size traits in WB chicken. CB chest breadth; CD Chest depth; TH Thoracic horn.


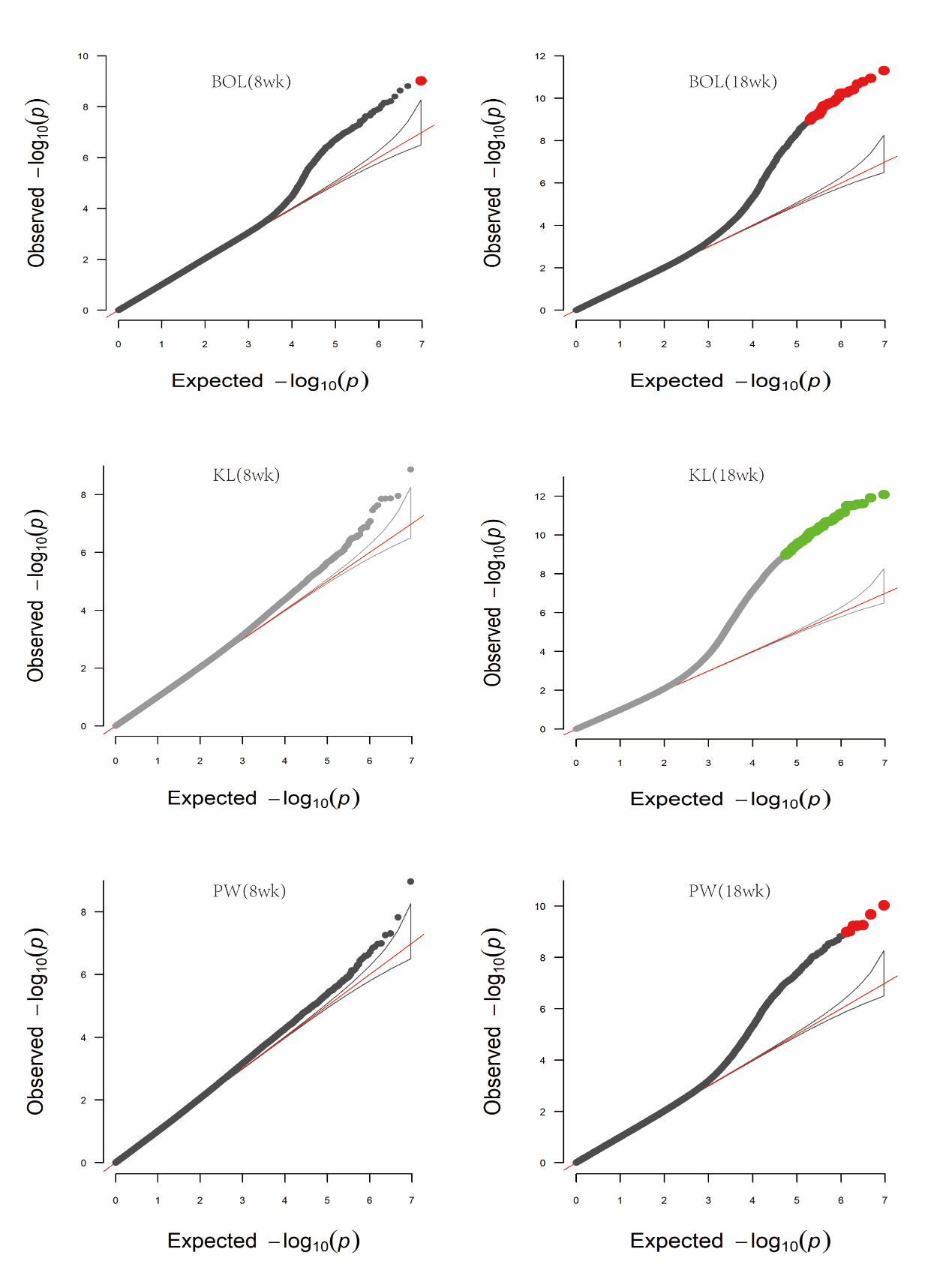


Fig.S4 QQ plots of GWAS for body size traits in WB chicken. BOL Body oblique length; KL Keel length; PW Pelvic width.


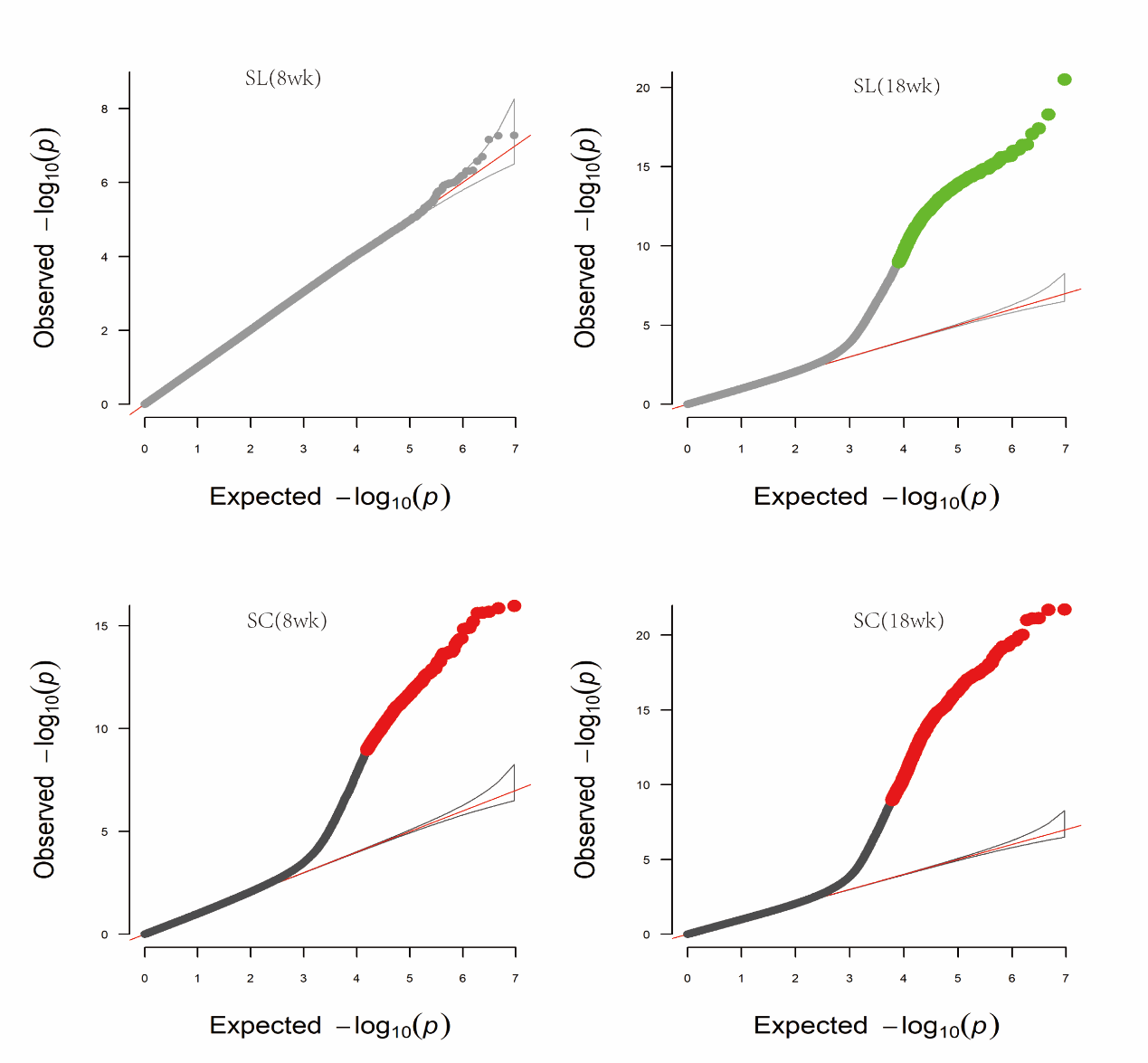


Fig.S5 QQ plots of GWAS for shank length and circumference traits in WB chicken. SL shank length; SC shank circumference.
